# Identifying mechanisms that underlie links between *COMT* genotype and aggression in male adolescents with ADHD

**DOI:** 10.1111/jcpp.12464

**Published:** 2015-09-23

**Authors:** Stephanie H.M. van Goozen, Kate Langley, Clare Northover, Kelly Hubble, Katya Rubia, Karen Schepman, Michael C. O'Donovan, Anita Thapar

**Affiliations:** ^1^ School of PsychologyCardiff UniversityCardiffUK; ^2^ MRC Centre for Neuropsychiatric Genetics and GenomicsCardiff UniversityCardiffUK; ^3^ Institute of Psychiatry, Psychology and NeuroscienceKings College LondonLondonUK; ^4^ Institute of Psychological Medicine and Clinical NeurosciencesCardiff University School of MedicineCardiffUK

**Keywords:** ADHD, aggression, conduct disorder, COMT, genetic, child

## Abstract

**Background:**

There is a known strong genetic contribution to aggression in those with ADHD. In a previous investigation of a large population cohort, impaired ‘emotional/social cognitive’ processing, assessed by questionnaire, was observed to mediate the link between *COMT Val158Met* and aggression in individuals with ADHD. We set out to replicate and extend this finding in a clinical sample, using task‐based and physiological assessments of emotional and cognitive processing. Our aim was to test the hypothesis that directly assessed emotional processing mediates the link between *COMT Val158Met* and aggression in young people with ADHD.

**Methods:**

Males aged 10–17 years with ADHD were recruited from UK community clinics (*n* = 194). Research diagnostic interviews (parent and child) were used to assess psychopathology and generate DSM‐IV Conduct Disorder symptom scores. Participants completed tasks assessing executive function (response inhibition and set shifting), empathy for fear, sadness and happiness, and fear conditioning [measured using skin conductance responses (SCR) to aversive stimuli].

**Results:**

*COMT* V*al* allele carriers showed poorer response inhibition (*F* = 5.27, *p* = .02) and set shifting abilities (*F* = 6.45, *p* = .01), reduced fear empathy (*F* = 4.33, *p* = .04) and reduced autonomic responsiveness (lower SCRs) to the conditioned aversive stimulus (*F* = 11.74, *p* = .001). *COMT Val158Met* did not predict impairments in recognising others' emotions or affective empathy for happiness or sadness. Mediation analysis revealed that impaired fear‐related mechanisms indirectly mediated the link between *COMT Val158Met* and aggression.

**Conclusion:**

Our findings suggest fear mechanisms as possible targets for psychological interventions to disrupt links between genetic risk and aggressive outcomes in ADHD. Our findings also reveal the potential of hypothesis‐driven approaches for identifying neuropsychological mechanisms that mediate genetic risk effects on behaviour and psychopathology.

## Introduction

Aggression is a well‐established adverse, potentially harmful outcome in individuals with ADHD (Klein et al., 2012; Thapar, Langley, Owen, & O'Donovan, 2007). It also indexes an important ADHD clinical subtype characterised by greater clinical severity and poorer prognosis even after treatment (Klein et al., 2012; Thapar et al., 2007)^.^ Thus, it is a priority to identify novel psychological and biological targets for informing the future development of effective interventions. However, to achieve this goal, we need to identify causal mechanisms that underlie the development of aggression in patients with ADHD.

Genetic vulnerability is one important and established contributor as there is consistent evidence that increased levels of aggression/conduct problems in ADHD index higher familial recurrence risk of ADHD (Faraone, Biederman, & Monuteaux, 2000), higher heritability (Thapar, Harrington, & McGuffin, 2001) and a greater burden of ADHD molecular genetic risk variants (Hamshere et al., 2013). Although there is consistent evidence that genes contribute to aggression in children with ADHD, the mechanisms by which they confer risk remain unknown. Increasingly, gene variants are presumed to impact on psychopathology by influencing processes at multiple levels including those that are neuropsychological as well as neural and molecular (Rasetti & Weinberger, 2011). However, little is known about the function of most individual genes and gene variants, so at present identifying risk mechanisms is a challenge. Nevertheless, there are some gene variants that have been extensively investigated in human and animal studies.

*COMT Val158Met* is a single nucleotide polymorphism within the gene encoding the enzyme catechol‐O‐methyl transferase. This enzyme breaks down catecholamines and is the primary mechanism by which dopamine is cleared in the prefrontal cortex. The gene variant *COMT Val158Met* affects enzyme activity levels with the *Val* variant breaking down dopamine four times faster than the *Met* variant. It is a gene variant that has been very well researched in human and animal studies. Meta‐analyses show that *COMT Val158Met* has pleiotropic effects on both executive function and emotional measures (Mier, Kirsch, & Meyer‐Lindenberg, 2009), processes that are important in relation to aggression, as well as effects on antisocial behaviour in those with ADHD (Caspi et al., 2008). Studies reveal worse performance on cognitive tasks assessing executive function in high‐activity *Val* allele carriers (Barnett et al., 2007; Langley, Heron, O'Donovan, Owen, & Thapar, 2010; Tunbridge, Harrison, & Weinberger, 2006). The *Val158Met* polymorphism is also associated with performance on paradigms involving emotional arousal and fear processing (Mier et al., 2009; Montag et al., 2008). These findings are remarkably similar in mice (Papaleo et al., 2008).

**Figure 1:**
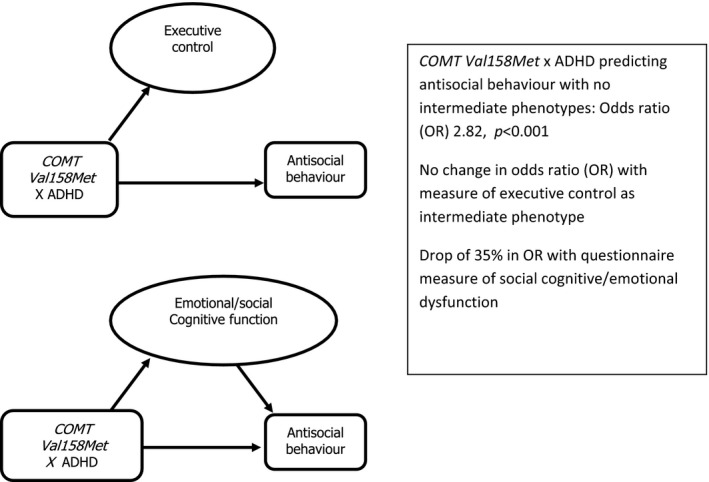
*COMT Val158Met* in a population cohort predicting antisocial behaviour/aggression in those with higher levels of ADHD with and without intermediate phenotypes (modified from Langley et al., 2010)

In a previous investigation of *COMT Val158Met* in a large, healthy population cohort of 4,365 children (Langley et al., 2010), findings suggested that social/emotional functioning mediated the link with aggression/antisocial behaviour in those with higher ADHD scores. In keeping with findings from previous pooled analyses (Caspi et al., 2008; Mier et al., 2009), this gene variant was found to predict three phenotypes: a task assessing executive control, a questionnaire measure of social/emotion function and aggression/antisocial behaviour in children with higher ADHD scores. However, the questionnaire measure of social/emotional function rather than executive control appeared to partially mediate the link between COMT genotype and aggression/antisocial behaviour in children with higher ADHD scores (see Figure 1).

In the present study, we set out to replicate and extend this finding and more precisely delineate the link in a patient sample. Rather than using questionnaires to measure emotional processing, we conducted lab‐based cognitive and physiological assessments of empathy and fear conditioning as well as executive functioning in a clinical sample of adolescent males with ADHD (*n* = 194). Our aim was to replicate and extend previous findings and test the hypothesis that emotional processes characterised at a behavioural and physiological level bridge the link between *COMT* and aggression in children with ADHD.

## Method

### Sample

Participants were boys aged 10–17 years with DSM‐IV (American Psychiatric Association, 2000) ADHD or ICD‐10 (World Health Organisation, 1993) Hyperkinetic Disorder (full criteria during childhood), as confirmed by a research diagnostic interview and were recruited from child and adolescent mental health or community paediatric clinics in Wales, UK. Most had participated in a previous, much larger genetic study and initial assessment details are described in full elsewhere (Hamshere et al., 2013). In accordance with DSM‐IV used at that time, children with schizophrenia, bipolar disorder, autism, Tourette's syndrome, neurological or genetic disorder were excluded. ADHD pervasiveness across settings was confirmed using the Child ADHD Teacher Telephone Interview (see Hamshere et al., 2013). In total, 194 adolescent males were genotyped and took part in the present study (mean age = 13.95 years, standard deviation 1.82 years). Ethics approval was obtained from the Wales Multicentre Research Ethics Committee. After complete description of the study to the subjects, written informed consent was obtained from parents and adolescents aged over 16 years. Written assent was obtained for younger adolescents.

### Clinical measures

Child psychopathology was reassessed using the Development and Well Being Assessment (DAWBA) structured interview using parents and adolescents as informants (Goodman, Ford, Richards, Gatward, & Meltzer, 2000). Parents completed the ADHD and Conduct Disorder (CD) sections and adolescents the Conduct Disorder section. All interviews were administered by trained psychologists supervised by an experienced clinician (AT). Symptom scores and diagnoses were generated according to DSM‐IV criteria. CD symptoms were counted as present when endorsed by either the parent or adolescent to generate DSM‐IV diagnoses of CD. In view of previous genetic findings showing associations are driven by aggression items (Hamshere et al., 2013; Monuteaux, Biederman, Doyle, Mick, & Faraone, 2009), we summed the subset of aggressive DSM‐IV CD items to generate a quantitative measure using the same approach (see Table S1 available online and DSM‐IV for symptom frequency and duration requirements). Additional psychopathology including adolescent and parent‐rated emotional/anxiety symptoms were assessed using the Strengths and Difficulties Questionnaire (SDQ) completed as part of the DAWBA (Goodman et al., 2000) rather than by interview to reduce the testing burden for participants. The five emotional items were summed to obtain a total emotional symptom score. IQ scores of >70 were established using the Wechsler Abbreviated Scale of Intelligence (Wechsler, 1999) – two‐subset form (vocabulary and matrix reasoning). No participant was stimulant naive. Those who continued to take stimulant medication (71%) were asked to discontinue 24 hr prior to testing.

### Genotyping

DNA was extracted from saliva (Oragene) or venous blood samples. Genotype data for the *COMT Val158Met* SNP (rs 4680) had been generated on most previous participants using the GWAS Illumina 660K array (Stergiakouli et al., 2012). For the remainder who had not been genotyped, genotyping was performed using Snapshot single‐base extension assays (Life Technologies, Carlsbad, CA). For details of the primers and specific protocol, please see http://psych.cf.ac.uk/home2/langley/COMT%20Genotyping%20Protocol.docx For additional information on genotypes, please see supplementary material (Appendix S1).

### Executive function tasks

Task selection was informed by previous findings that have shown association between *COMT Val158Met* and performance on the Wisconsin Card Sorting Task (Heaton, 2005) as well as a response inhibition task in healthy children (Langley et al., 2010).

#### Wisconsin card sorting task

The Wisconsin Card Sorting Task (WCST) (Heaton, 2005) measures the ability to display flexibility in the face of changing schedules of reinforcement (‘set shifting’). It is considered to provide an indicator of prefrontal cortical functioning and involves the participant having to sort and match cards on the basis of criteria that change and that have to be worked out by using feedback on whether the match is correct or not. We used two measures of task performance; total number of errors and perseverative errors.

#### Go/No‐Go task

The GNG task (Go/No‐Go) (Rubia, Smith, & Taylor, 2007) measures motor response inhibition, a domain shown to be consistently impaired in ADHD (Hart, Radua, Nakao, Mataix‐Cols, & Rubia, 2013). The participant is required to use their dominant hand to either respond or inhibit their response depending on whether there is a Go (a spaceship, 73% of trials) or a No‐Go (a planet, 27% of trials) target. The task was administered in a block of 150 trials. The selected measure of task performance was the percentage of successful inhibitions to the No‐Go targets.

### Emotional/social processes

Tasks were selected to assess emotional and social cognitive processes which were judged to best reflect processes indexed by the Social and Communication Disorders Checklist (SCDC) that was utilised in the Langley et al. (2010) cohort analysis.

#### Cognitive and affective empathy

Three clips depicting the emotions of sadness, happiness and fear were edited from cinematic films (van Rijn, Barendse, van Goozen, & Swaab, 2014). Given previous findings (e.g. Jones, Happe, Gilbert, Burnett, & Viding, 2010) highlighting the dissociation of empathy into ‘feeling’/‘resonating’ with another's emotions (affective empathy) versus correctly identifying emotions in others (cognitive empathy), both constructs were assessed using previously published methods (see Van Rijn et al., 2014). After each clip participants completed a questionnaire concerning the recognition of the emotions of the main character (cognitive empathy) and their own emotions while viewing the clip (affective empathy), they were also asked to explain the reason for the emotion (‐s) identified in the main character and themselves. These responses were coded separately for cognitive and affective empathy using a scoring system which took into consideration four elements of empathy: (a) the correct target emotion, (b) other similar and relevant emotions, (c) the intensity of the emotion, and (d) the explanation for the causes of the emotion. Cognitive and affective empathy scores were calculated for each clip and ranged from 0 to 6 (van Rijn et al., 2014). For both scales, a higher score is indicative of greater empathy. Internal consistency for cognitive and affective empathy was .62 and .79 respectively; interscorer reliabilities for the both measures between two blind raters using a subset of the data (10%) across the three film clips ranged from .74 (cognitive empathy) to .82 (affective empathy).

#### Fear conditioning assessed using skin conduct response

Skin conductance response (SCR) involves assessing electrical conductance that is affected by sympathetic nervous system activity. Electrodermal activity was recorded using a skin conductance amplifier (PSYCHLAB Contact Precision Instruments, UK) and sampled at 500 Hz from the distal phalanges of the index and middle fingers of the nondominant hand. The fear conditioning paradigm replicated the procedure described by Bechara and Damasio (2002) and Fairchild, Van Goozen, Stollery, and Goodyer (2008). Participants view 48 coloured slides (red, blue, orange, green) presented on a computer screen. The blue slides are randomly presented and paired with a loud (99 dB) aversive white noise lasting 1,000 msec presented bi‐aurally using headphones. The slides serve as the visual conditioned stimuli (CS), the aversive loud noise is the unconditioned stimulus (US), and SCRs to the CS+ (unreinforced blue slide) and CS− (red slide) are the dependent variables. In accordance with previous studies, valid SCRs exceed an amplitude of .05 *μ*Siemens (*μ*s) (Fairchild et al., 2008). The internal consistencies for the SCRs to the US and CS+ were 0.81 and 0.70 respectively.

### Data analyses

Analysis of variance (ANOVAs) was used to examine differences in task performance by *COMT Val158Met* genotype (Met/Met vs. Val carrier) and according to the presence/absence of categorically defined aggression (ADHD vs. ADHD+CD). For the fear conditioning paradigm, measures were mean SCRs to the CS+ (unreinforced blue slides) or CS− (red slides), or mean increase in SCRs during the CS+ or CS− corrected for baseline SCRs. Correlations were assessed for dimensional measures using Pearson's correlation coefficient. Analyses were carried out using SPSS 20 (SPSS Inc., Chicago, IL).

Depending on the results of our primary analyses, we planned to test significantly associated task measures as mediator variables on the pathway between *COMT Val158Met* genotype and aggression in ADHD. Mediation analyses were undertaken according to the criteria proposed by MacKinnon, Lockwood, Hoffman, West, and Sheets (2002). This approach enables statistical tests of the indirect pathway (x ‐> m ‐> y) where the independent variable (*COMT Val158Met* genotype; x) and dependent variable (the dimensional measure of aggression symptom score; y) are both significantly associated with the putative mediator (e.g. executive function or fear conditioning; m). Significance of the indirect pathway was considered if the bias corrected 95% confidence interval did not cross zero (Preacher & Hayes, 2008).

## Results

### Executive function and emotion processing task performance by COMT genotype

In accordance with previous papers, the *Val/Val* and *Val/Met* genotype groups were collapsed into one group. Table 1 shows demographic and clinical data for the sample by genotype group (Table S2 provides mean scores for the three genotype groups). The genotype groups differed by age, so age was included as a covariate in subsequent analyses. IQ was included as a second covariate as lower IQ (but no other clinical or demographic variable) was associated with higher levels of aggressive behaviour (defined as a diagnosis and quantitative score; the IQ difference for those who met DSM‐IV criteria for Conduct Disorder versus no Conduct Disorder, *F*[1,196] = 17.98, *p* < .001).

**Table 1:** Demographic and clinical characteristics by *COMT* genotype group, mean scores (standard deviation)

	Met/Met	Val/Val &Val/Met	*p*
IQ	85.9 (9.3)	88.1 (10.0)	.16
Age	14.4 (1.7)	13.7 (1.8)	.02
ADHD symptom severity	12.9 (4.0)	12.3 (4.9)	.43
SDQ^a^ Anxiety symptoms	4.8 (2.9)	5.0 (2.7)	.74

All between group analyses were done using one‐way ANOVAs.

a Strength and Difficulties Questionnaire self‐rated.

Comparison of the genotype groups (Table 2) showed significant differences in performance on tasks assessing executive function, affective empathy for fear and fear conditioning. Carriers of the *Val* allele had poorer response inhibition and set shifting abilities (less cognitive flexibility as reflected by more total and perseverative errors), reduced fear empathy (feeling someone else's fear) and more impaired fear conditioning (reduced SCR increase to the CS+). *COMT Val158Met* did not predict impairments in recognising others' emotions (cognitive empathy) or the vicarious experience of others' happiness or sadness; effects were restricted to fear empathy. The number of participants differed between individual tasks due to practical (task not fully completed) and experimental (data not useable) considerations, but there were no significant differences on clinical, demographic or genotypic measures between those who did and did not complete each task (see Table S3) or between those on medication versus not.

**Table 2:** Task performance mean scores (standard deviations) by genotype group

	Met/Met	Val/Val and Val/Met	*F* value	*p*
Executive functioning				
WCST^a^ ^total errors^	16.4 (7.2)	19.3 (9.6)	3.98	.048
WCST^perseverative errors^	7.6 (4.1)	10.1 (6.6)	6.45	.012
GNG^b^ ^inhibition^	49.8 (18.5)	40.9 (19.4)	5.27	.023
Cognitive empathy				
Happy	4.8 (0.7)	4.7 (0.8)	0.34	.56
Sad	5.0 (0.6)	5.0 (0.5)	0.05	.83
Fear	5.0 (0.4)	5.1 (0.4)	0.41	.53
Affective empathy				
Happy	2.7 (1.9)	2.9 (1.8)	0.24	.62
Sad	3.0 (2.2)	2.9 (2.1)	0.11	.74
Fear	2.2 (2.2)	1.5 (1.9)	4.33	.039
Fear conditioning				
CS+^unreinforced blue slide^	0.30 (0.6)	−0.12 (0.5)	11.74	.001
CS−^red slide^	−0.20 (0.4)	−0.24 (0.6)	0.12	.73

All between group analyses were done using ANCOVAs correcting for age and IQ.

Number of participants per task: WCST (*n* = 165), GNG (*n* = 160), Cognitive and Affective empathy (*n* = 166), Fear conditioning (*n* = 108).

a Wisconsin Card Sorting Task assessing ability to shift strategy.

b Go/no‐Go Task assessing ability to inhibit a response.

*COMT Val158Met* did not directly predict aggression (mean score for Val allele carriers 1.2 (*SD* 1.4) versus 1.1 (*SD* 1.3); *p* = .83) or conduct disorder (mean conduct score for Val allele carriers 3.5 (*SD* 3.1) versus 3.3 (*SD* 2.8); *p* = .73; 48.6 % with CD diagnosis in Val allele carriers, *p* = .73) in this sample in contrast to the original total larger genotyped ADHD sample from which the participants were drawn.

### Executive function and emotion processing associations with aggression

Comparison between ADHD adolescents with and without a diagnosis of Conduct Disorder (CD) revealed no group differences in executive performance or cognitive empathy; similarly, the quantitative measure of aggression was not associated with measures of executive functioning or cognitive empathy (see Table 3). However, those with greater levels of aggression (CD vs. no CD; higher aggression scores) showed lower affective empathy scores (for fear, happy and sad emotions) and reduced autonomic responsiveness to the CS+ (lower skin conductance responses, SCRs) (see Table 3).

**Table 3:** Differences between subgroups with and without CD, and associations between task performance and aggression

	No CD^a^ Mean (*SD*)	CD Mean (*SD*)	No CD versus CD *F* (*p* value)	Correlation with aggression scores Pearson's *r* (*p* value)
Executive functioning				
WCST^total errors^	17.5 (8.9)	19.5 (9.0)	0.09 (.77)	0.12 (.10)
WCST^perseverative errors^	8.8 (5.8)	10.1 (6.5)	0.20 (.65)	0.11 (.14)
GNG^inhibition^	42.5 (20.3)	44.4 (18.6)	0.22 (.64)	−0.02 (.82)
Cognitive empathy				
Happy	4.7 (0.8)	4.7 (0.7)	0.01 (.96)	−0.08 (.28)
Sad	5.0 (0.5)	5.0 (0.5)	0.05 (.83)	0.03 (.67)
Fear	5.1 (0.4)	5.1 (0.4)	0.03 (.86)	0.07 (.37)
Affective empathy				
Happy	3.3 (1.8)	2.4 (1.8)	10.31 (.01)	−0.31 (<.001)
Sad	3.3 (2.1)	2.6 (2.1)	3.49 (.06)	−0.22 (<.01)
Fear	2.1 (2.0)	1.4 (1.9)	5.45 (.02)	−0.26 (<.001)
Fear conditioning				
CS+^unreinforced blue slide^	0.17 (0.4)	−0.01 (0.3)	5.97 (.016)	−0.27 (<.01)
CS−^red slide^	−0.27 (0.3)	−0.17 (0.4)	3.37 (.07)	0.06 (.56)

All between group analyses were done using ANCOVAs correcting for Age and IQ.

Number of participants per task: WCST (*n* = 165), GNG (*n* = 160), Cognitive and Affective empathy (*n* = 166), Fear conditioning (*n* = 113).

a Conduct Disorder.

### Assessing mediation

**Figure 2:**
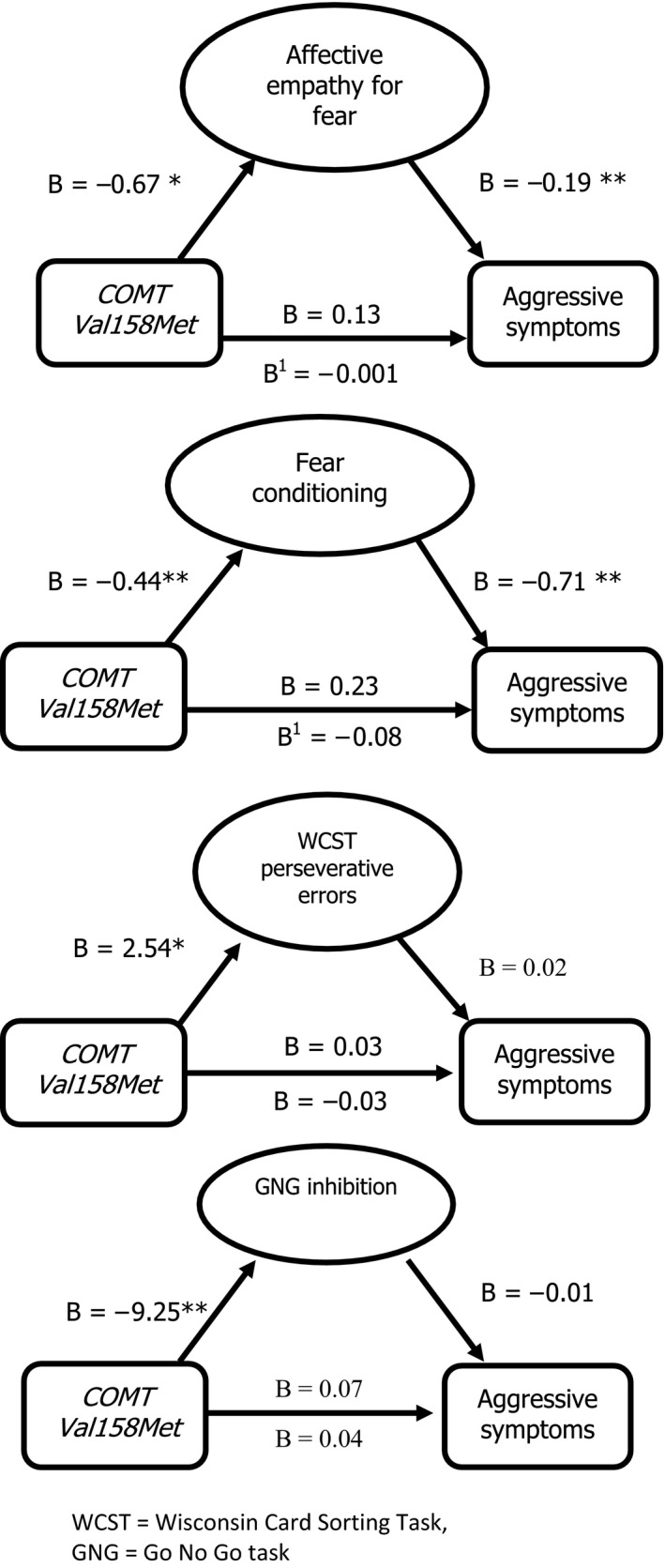
Testing performance on fear‐related and executive functioning tasks as mediators in a clinical sample. WCST, Wisconsin Card Sorting Task; GNG, Go/No‐Go task

Mediation analysis was conducted using *Val* carrier status as the predictor and aggression scores as the outcome and included in the model (a) a direct pathway between predictor and outcome and (b) an indirect pathway via the ‘intermediate’ variables of (i) fear empathy and (ii) fear conditioning. Two separate analyses were conducted as performance on the task assessing affective empathy for fear and the physiological measure of fear conditioning (SCR increase to CS+) were not significantly associated although a trend towards association was observed (*r* = .19, *p* = .07). Neither affective measure was associated with any of the executive function measures (*p* > .10; results available from authors). Mediation analyses showed that the bias corrected confidence intervals for the path coefficients did not cross zero for both models (0.091–0.613 for fear conditioning; 0.095–0.288 for fear empathy), consistent with a significant indirect effect of *COMT* genotype on aggression (Preacher & Hayes, 2008) through the two different measures involving fear processing‐ fear empathy and fear conditioning (Figure 2; results for executive functioning also shown for comparison).

## Discussion

The results of this study once again show pleiotropic effects of *the COMT Val158Met* polymorphism on executive function and emotion. This has been observed in healthy adults and young people (Langley et al., 2010; Mier et al., 2009), patients with schizophrenia (Tunbridge et al., 2006) and in mice (Papaleo et al., 2008) and extends to male adolescents with ADHD. Specifically *Val* carriers showed poorer set shifting abilities and response inhibition (measures of executive function), poorer fear‐related empathy and greater failure to condition to an aversive stimulus (CS+) as assessed by skin conductance responses. We found that in this ADHD sample, measures of executive function did not predict aggressive outcomes, whether defined categorically in terms of clinical diagnosis or dimensionally as aggressive scores, whereas affective empathy, that is response to others' emotions (fear, happiness and sadness) and reduced autonomic responsiveness in the conditioning experiment did. This was not explained by impaired ability to understand others' emotions (cognitive empathy). Mediation analysis including the intermediate phenotypes indicated that the *Val* allele had a significant indirect effect on aggression via emotional (fear empathy and fear learning) rather than executive control mechanisms.

Previous findings shaped the present investigation and our hypotheses (Langley et al., 2010). In a large UK population cohort data set of healthy children with higher levels of ADHD symptoms, we had observed that while *COMT Val158Met* predicted aggression/antisocial behaviour, performance on the available measure of executive function and a questionnaire measure capturing emotion/social cognitive items, the link between genotype and aggression was mediated via the measure of emotional/social cognitive function. The present study focused on patients and utilised static and dynamic task‐based assessments of emotion/social cognitive processes of the type judged to be captured in the original questionnaire. The findings not only suggest it is emotional processes rather than executive dysfunction that mediate links between genotype and aggression but also more specifically that these involve differences at a physiological level as well as in terms of reported experiences and appear to be specific to the emotion of fear. In the previous cohort study (Langley et al., 2010), and in a meta‐analysis (Caspi et al., 2008) *COMT Val158Met* predicted antisocial behaviour in those with ADHD. Here, although we observed genetic association with performance on three different types of neuropsychological/physiological task, no direct association with aggression was observed. Links with aggression were indirectly mediated. *COMT Val158Met* has been observed to predict aggression/extreme antisocial behaviour in individuals with ADHD (Caspi et al., 2008; Langley et al., 2010; Monuteaux et al., 2009; Salatino‐Oliveira et al., 2012) across six independent studies and in a meta‐analysis (Caspi et al., 2008) with the association applying to the subtype of aggression in those with ADHD, not to aggressive behaviour in the general population (Caspi et al., 2008). Given the small effect size of *COMT Val158Met* observed in the original studies and meta‐analysis, the most plausible reason for failing to observe a direct effect in this subsample is that the sample size was too small.

The relationship between *COMT Val158Met* and physiological fear conditioning paradigms is beginning to be examined also in healthy adults and adults with anxiety disorders and posttraumatic stress disorder (PTSD) (Norrholm et al., 2013). They suggest that Val carriers versus Met/Met genotype show reduced startle in response to fear stimuli (Montag et al., 2008) and lower fear memory consolidation (e.g.Lonsdorf & Kalisch, 2011). Our findings were not explained by lower levels of anxiety symptoms (as assessed by parent or adolescent rated SDQ) in the Met/Met group (see Table 1). Fear conditioning paradigms are used widely across different species to assess the important basic learning mechanism for distinguishing threat and safety (Pine, 2010). Impairment in this form of associative learning means that individuals do not learn to associate particular situations, behaviours and contexts with punishment and this has important implications for interventions that rely on this ability (Raine, 1993). Our results further suggest that poorer ability to feel other's fear in those with ADHD maybe a pathway from *COMT* to aggression. The importance of empathy skills in relation to psychopathology has become increasingly recognised and is an emerging area of neuroscience (Panksepp & Panksepp, 2013); empathy deficits have been proposed as an explanatory mechanism underlying social impairments characteristic of many different psychiatric disorders including Conduct Disorder and Autism Spectrum Disorders (ASD)(Bons et al., 2013). The present study supports findings that understanding (cognitive empathy) and feeling (affective empathy) others' emotions can be meaningfully disaggregated (Bons et al., 2013).

Our findings have potentially important clinical implications. Current approved treatments of ADHD as well as medication include behavioural interventions that are based on social learning theory (NICE, 2008). Thus far, long‐term benefits of such treatments in terms of aggressive outcomes in those with ADHD have not been demonstrated (Molina et al., 2009). If impaired fear empathy and fear learning are critical risk mechanisms, then existing behavioural and psychosocial interventions for ADHD will have to be adjusted or enhanced for some individuals to address these deficits.

There is growing interest in defining basic dimensions of functioning at cognitive, physiological and molecular levels as exemplified in the NIMH Research Domain Criteria RDoC (Insel et al., 2010). One aim in doing so is to better explain how genetic vulnerability leads to clinically observable behaviour. It is hoped that using such an approach may help identify risk mechanisms that can be used to subtype psychiatric disorders. To our knowledge, evidence of this being a fruitful approach has yet to be empirically demonstrated. Our findings do indeed suggest that characterising patients using dimensional static and dynamic task‐based measures, including physiological ones such as fear conditioning, can help disaggregate links between genetic risk and behavioural/psychiatric outcomes and identify risk mechanisms. Future imaging studies will be required to further identify risk mechanisms at the level of brain circuitry. However, here we had an a priori rationale for focusing on a functional gene variant that has been very well researched thereby enabling a hypothesis‐driven approach.

There are some limitations to our study. Some data could not be used, some were missing. This is to be expected given subjects had ADHD and were taken off medication. However, there were no clinical or genotype differences between those whose data were and were not available (see Table S3), we adjusted for age and IQ, and the findings were not explained by comorbid anxiety/emotional problems. As these were typical adolescent clinic patients, none were drug naive and treatment could have reduced symptom levels in some thereby reducing power to detect associations with aggression. Our sample size while much larger than previous lab‐based cognitive and psychophysiological studies of adolescent patients, was small for a genetic investigation and included males only because of known *COMT* sexual dimorphism (Tunbridge & Harrison, 2011). However, we were still able to demonstrate indirect effects of genotype via reduced fear conditioning and fear empathy on aggression in ADHD using mediation analysis. As our hypothesis involved ADHD patients only [*COMT Val158Met* does not predict aggression in the general population (Caspi et al., 2008)], for the present investigation we did not examine healthy controls. Future studies will be required to further examine healthy adolescents and the development of aggression in other populations and to replicate these findings. Finally, although all analyses were hypothesis‐driven, the inclusion of tasks, genotype and clinical outcome meant conducting multiple tests. Replication will be required.

Longitudinal population studies have shown that impaired fear conditioning in early childhood (in those without ADHD) may predict involvement in antisocial behaviour in the general population (Gao, Raine, Venables, Dawson, & Mednick, 2010), whereas enhanced conditioning may act as a protective factor. Although we found that specific emotional processes were associated with aggression it is not known whether these – or similar deficits – preceded the occurrence of aggression (and might be causal) or if they represent a consequence of it. Another possibility is that we are observing pleiotropic effects of the *Val158Met* polymorphism on outcome and mediators, which cannot be disaggregated from causal links using a cross‐sectional design. Future prospective studies need to establish whether the observed emotional mechanisms explain the emergence and worsening of aggression in ADHD. There will also be a need to test the contribution of other genetic and nongenetic risk factors.

In conclusion, the present study suggests that in male adolescents with ADHD fear‐related processes, defined both behaviourally and physiologically, might indirectly mediate links between *COMT Val158Met* genotype and clinically reported aggression. These findings highlight the potential of hypothesis‐driven investigations of key neuropsychological mechanisms that mediate genetic risk effects on behavioural outcomes and psychopathology.

**Key points**

- Previous research suggests impaired socioemotional processing, assessed by questionnaire, rather than executive control mediates the link between a gene variant *COMT Val158Met* and aggression in individuals with ADHD.
- In a clinical sample of adolescent males with ADHD, we find that lower empathy for other's fear and fear learning (using a fear conditioning paradigm) but not executive control mediate the link between *COMT Val158Met* and aggression.
- Our findings suggest fear‐related processes might represent important links between genetic risk and aggressive outcomes in ADHD.
- Psychological interventions that address fear‐related processes could help establish whether this is one route by which genetic risk of aggression in those with ADHD can be reduced.

## Supporting information


**Table S1**. DSM‐IV aggressive conduct disorder symptoms.
**Table S2**. Mean aggression, executive function, fear empathy and fear conditioning scores.
**Table S3**. Comparisons of included and excluded cases [mean scores (standard deviations)] on different types of tests.
**Appendix S1**. Further information on genotyping.
