# Supplementary material for: Identifying mechanisms that underlie links between COMT genotype and aggression in male adolescents with ADHD
Source: J Child Psychol Psychiatry. 2015 Sep 23;57(4):472–80. doi: 10.1111/jcpp.12464 (PMC5102627; doi:10.1111/jcpp.12464)
Supplement: Supplementary file 1 — Table S1. DSM‐IV aggressive conduct disorder symptoms. Table S2. Mean aggression, executive function, fear empathy and fear conditioning scores. Table S3. Comparisons of included and excluded cases [mean scores (standard deviations)] on different types of tests. Appendix S1. Further information on genotyping. [file JCPP-57-472-s001.docx]

**Supporting information for *Identifying mechanisms that underlie links between COMT genotype and aggression in male adolescents with ADHD* by Thapar et al.**

**Table S1:** DSM-IV aggressive conduct disorder symptoms

| **Aggressive symptoms** |
| --- |
| Often bullies, threatens or intimidates others  Often initiates physical fights (not including fights with siblings)  Has used weapon that can cause serious physical harm to others  Has been physically cruel to people  Has been physically cruel to animals  Has stolen while confronting the victim (e.g. mugging, purse snatching, extortion)  Has forced someone into sexual activity |

| **Table S2:** Mean aggression, executive function, fear empathy and fear conditioning scores in children with ADHD according to each of the three *COMT Val158Met* genotypes | | | | | | | |
| --- | --- | --- | --- | --- | --- | --- | --- |
|  | Met/Met | | Val/Met | | Val/Val | |  |
|  |  |  |  |  |  |  |  |
|  | Mean | *S.D.* | Mean | *S.D.* | Mean | *S.D.* | p |
| Aggression score | 1.1 | *1.3* | 1.1 | *1.2* | 1.4 | *1.7* | 0.57 |
| WCST ^total errors^ | 16.6 | *7.0* | 18.1 | *8.9* | 20.7 | *10.2* | 0.052 |
| WCST ^perseverative errors^ | 7.6 | *4.1* | 9.7 | *6.4* | 10.6 | *6.9* | 0.039 |
| GNG ^inhibition^ | 49.6 | *18.6* | 40.8 | *19.1* | 41.5 | *20.0* | 0.10 |
| Fear - Affective empathy | 2.2 | *2.2* | 1.6 | *1.9* | 1.5 | *1.9* | 0.084 |
| Fear conditioning CS^+^ | 0.31 | *0.58* | -0.03 | *0.51* | -0.25 | *0.55* | <0.001 |

*Original hypothesis was based on two genotype groups-this is provided for descriptive purposes*

*All between group analyses were done using one-way ANOVAs corrected for age and IQ*

*Number of participants per task: WCST (n=165), GNG (n=160), Affective empathy for fear (n=166), Fear conditioning (n=108).*

**Table S3:** Comparisons of included and excluded cases (mean scores [standard deviations]) on different types of tests

*Executive Functioning*

|  | **Included EF** | **Excluded EF** | **P** |
| --- | --- | --- | --- |
| IQ | 87.5 (10.3) | 84.6 (8.9) | Ns |
| Age | 13.9 (1.8) | 14.4 (1.8) | Ns |
| ADHD symptoms | 12.3 (4.7) | 13.5 (3.4) | Ns |
| Aggressive symptoms | 1.1 (1.3) | 1.4 (1.6) | Ns |

*Empathy*

|  | **Included empathy** | **Excluded empathy** | **P** |
| --- | --- | --- | --- |
| IQ | 87.7 (9.4) | 83.2 (14.4) | Ns |
| Age | 13.9 (1.8) | 14.1 (1.7) | Ns |
| ADHD symptoms | 12.3 (4.7) | 14.2 (3.3) | Ns |
| Aggressive symptoms | 1.1 (1.3) | 1.6 (1.5) | Ns |

*Fear conditioning*

|  | **Included conditioning** | **Excluded conditioning** | **P** |
| --- | --- | --- | --- |
| IQ | 87.4 (10.3) | 87.6 (9.6) | Ns |
| Age | 14.1 (1.6) | 13.7 (2.0) | Ns |
| ADHD symptoms | 12.4 (4.3) | 12.4 (5.4) | Ns |
| Aggressive symptoms | 1.2 (1.4) | 1.2 (1.3) | Ns |

*All between group analyses were done using one-way ANOVAs*

**Appendix S1: Further information on genotyping**

Genotyping was undertaken in n=177 individuals on both the Illumina and SNAPshot platforms with 97.7% concordance. Those with discordant data were excluded.

The total ADHD sample was in Hardy Weinberg Equilibrium. Chi square =0.48, df 1, p=0.49. The initial phase of laboratory-based psychology assessments involved selective genotype recall but later assessments were extended to the full sample.
